# Accelerating Hierarchical ZSM‑5 Engineering via Bayesian Optimization-Guided Discovery

**DOI:** 10.1021/acsmaterialsau.5c00196

**Published:** 2025-12-22

**Authors:** Tzu-Hung Wen, Cheng-Yi You, Ting-Hao Liu, Bryan R. Goldsmith, Yu-Chuan Lin

**Affiliations:** † Department of Chemical Engineering, 34912 National Cheng Kung University, Tainan 70101, Taiwan; ‡ Department of Chemical Engineering, 1259 University of Michigan, Ann Arbor, Michigan 48109-2136, United States; § Center for Resilience and Intelligence on Sustainable Energy Research (RiSER), 34912 National Cheng Kung University, Tainan 70101, Taiwan

**Keywords:** Active Learning, Bayesian Optimization, Catalysts, Hierarchy, ZSM-5

## Abstract

Bayesian optimization (BO) was used to accelerate the synthesis of hierarchical ZSM-5 with a balanced micro–mesoporous structure. Using mesoporosity and microporosity as dual objectives, a Gaussian process regression surrogate guided three successive BO iterations informed by 15 initial experiments. The optimized sample (HZ-R_0.55_T_50_t_2_) exhibited the highest hierarchy factor (0.17), featuring similar mesoporosity (0.44) but higher microporosity (0.38) than the benchmark, indicating reduced diffusional resistance with preserved framework. Characterizations revealed that NaOH induced framework dissolution, whereas TPAOH moderated desilication; their mixture synergistically created uniform mesopores while maintaining crystallinity. ^27^Al and ^29^Si NMR confirmed realumination and silanol generation, and acidity analysis showed redistributed acid sites. Sensitivity analysis identified the TPAOH fraction and temperature as the dominant factors influencing the hierarchy factor. This study establishes a data-driven workflow for zeolite design, demonstrating that BO effectively accelerates hierarchical structure optimization while minimizing the experimental effort.

## Graphical abstract

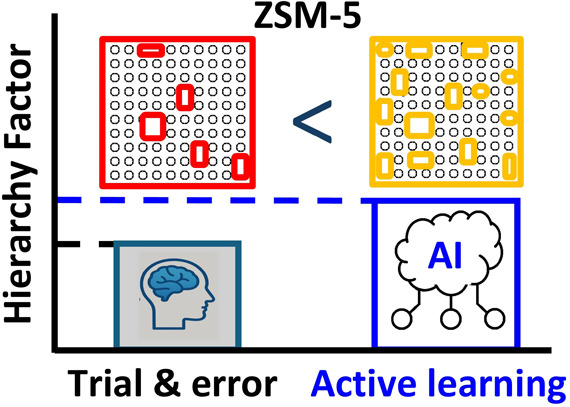

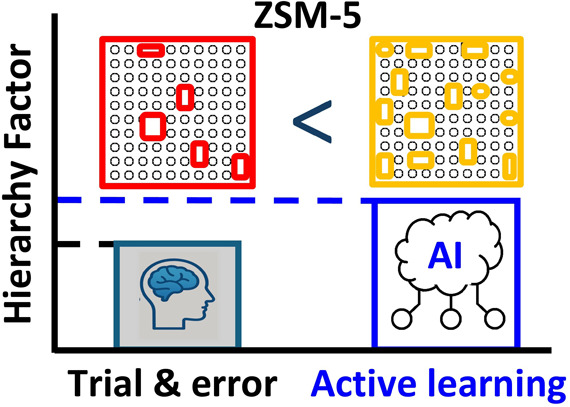

## 1 Introduction

ZSM-5 is a crystalline aluminosilicate zeolite with an MFI topology,^1^ which is widely used in naphtha cracking,^2^ methanol-to-hydrocarbon (MTH),^3^ and methanol-to-aromatic (MTA) reactions.^4^ Its channel architecture and acidity are key to achieving high product selectivity.^5^ Despite its broad applications, ZSM-5’s microporosity restricts transport of bulky molecules (e.g., aromatics) whose diameters exceed its pore openings.^6^ Its framework topology—comprising straight (5.1 × 5.5 Å) and zigzag (5.3 × 5.6 Å) channels forming a 3D network—exhibits high diffusional resistance and coke accumulation. Over the past several decades, strategies to extend catalyst lifetime in MTH have emerged,^7–10^ mainly by suppressing diffusion and accumulation of coke precursors within zeolite channels.^11–13^ Similar improvements have been reported for MTA^14–16^ and alcohol dehydration reactions.^17,18^

To reduce diffusional resistances, the development of hierarchical ZSM-5—featuring a combination of intrinsic micropores and secondary mesopores—has emerged as an effective strategy to enhance molecular transport by increasing the mesoporosity without significantly compromising microporosity.^19–21^ The dimensionless term called hierarchy factor (HF) was introduced as a quantitative metric to evaluate the balance between mesoporosity and microporosity.^22^ HF integrates relative micropore volume (*V*_micro_/*V*_Total_) and external surface areas (*S*_ext_/*S*_BET_) into a single figure of merit (eq 1), offering a practical guideline for optimizing hierarchical structures without significantly compromising the intrinsic microporosity of the parent zeolite.

1 $$HF=\frac{V_{micro}}{V_{Total}}\times \frac{S_{ext}}{S_{\mathrm{BET}}}$$

where *V*_micro_ is the micropore volume, *S*_ext_ is the external surface area, *V*_Total_ is the total pore volume, and *S*_BET_ is the surface area estimated by using the BET method.

Subsequent studies have demonstrated positive correlations between HF values and catalytic activity and product selectivity.^23–25^ Gil and co-workers^26^ reported that tailoring the hierarchical structure of ZSM-5 through adjusting NaOH concentration altered its Brønsted acidity. Varying NaOH concentration during ZSM-5 desilication enhanced aromatic yield and reduced coke formation in fast pyrolysis of beech wood.^27^ Li and co-workers^28^ examined the influence of tetraethylammonium hydroxide (TEAOH) concentration, finding that dealkylation products such as phenol and xylenes were sensitive to the amounts of TEAOH used in desilication of ZSM-5. Despite these advances, most studies still rely on the one-variable-at-a-time approach to optimize the ZSM-5 hierarchy.

Bayesian optimization (BO) has emerged as an efficient approach for optimizing synthesis and reaction conditions in catalysis and materials science.^29–31^ BO leverages surrogate models and acquisition functions to iteratively navigate the parameter space, significantly reducing the number of experiments needed to identify the optimum of an objective function.^32^ This approach is particularly advantageous for systems where experimental evaluations are expensive—such as the fine-tuning of postsynthetic zeolite treatments.

Herein, we apply BO to guide the desilication of hierarchical ZSM-5 to expedite the synthesis of materials with a balanced micro–mesoporous structure. Using mesoporosity and microporosity as objective functions, we mapped their correlation with desilication parameters to establish a data-driven framework. From 15 initial experiments and three successive BO iterations, an optimal condition was identified, yielding a higher HF value (0.17) than that of the benchmark material (0.13). The optimized sample exhibited comparable mesoporosity but greater microporosity, implying similar diffusional resistance yet a higher extent of framework preserved. This strategy highlights how data-guided desilication can suppress diffusional limitations while preserving the intrinsic acidity in hierarchized ZSM-5.

## 2 Experimental Section

ZSM-5 (CBV-8014, Si/Al = 40, Zeolyst International) was calcined in static air at 823 K for 5 h (5 °C min^–1^) to yield the protonated form (H^+^). Samples were designated HZ-*R*_*x*_ *T*_*y*_ *t*_*z*_, where *R* denotes the ratio of tetraethylammonium hydroxide (TPAOH) to the mixed sodium hydroxide (NaOH) and TPAOH solution [TPAOH]/([NaOH] + [TPAOH]) with *x* as the value of the ratio (*x* = 0–1.0), *T* as the treatment temperature (*y* = 50–90 °C), and *t* as the treatment time (*z* = 0.5–2 h).

### 2.1 Desilication Procedure

NaOH, TPAOH, and their mixtures were used as the desilication agents with the total hydroxide concentration fixed at 0.2 M.^22^ A Box–Behnken design within the response surface methodology (RSM) framework was applied to evaluate three input features: the TPAOH/(NaOH + TPAOH) ratio (*R*), desilication temperature (*T*), and treatment time (*t*). Fifteen conditions (Samples 1–15, Table 1) were used to construct the initial surrogate model for Bayesian optimization. The micropore (*V*_micro_/*V*_total_) and mesopore (*S*_ext_/*S*_BET_) ratios were defined as the objective functions to maximize the porosity development. *V*_micro_ is the micropore volume estimated by using the *t*-plot method, *V*_Total_ is the total pore volume obtained from the adsorbed N_2_ amount at *P*/*P*_0_ = 0.99, *S*_ext_ is the external surface area obtained from *S*_BET_ – *S*_micro_, where *S*_micro_ is the micropore surface area estimated by using the *t*-plot method, and *S*_BET_ is the surface area estimated by using the BET method. The hierarchical factor (HF) was not selected as an objective, as its multiplicative form can bias results; for instance, a high HF value may arise from a large micropore ratio combined with a low mesopore ratio, which does not reflect meaningful mesopore development.

**Table 1:** Porosity, Micropore Ratio, Mesopore Ratio, Hierarchy Factor (HF), and Relative Crystallinity (χ_c_) of ZSM-5 Samples with GPR-Predicted Values Shown in Parentheses

Sample	Denotation	*R* ^a^	*T* ^b^ (°C)	*t* ^c^ (h)	*V*_micro_ ^d^ (cm^3^ g^–1^)	*V*_Total_ ^e^ (cm^3^ g^–1^)	*S*_ext_ ^f^ (m^2^ g^–1^)	*S*_BET_ ^g^ (m^2^ g^–1^)	*V*_micro_/*V*_Total_	EI_micro_	*S*_ext_/*S*_BET_	EI_meso_	HF^h^	χ_c_ ^i^ (%)
	HZ-C	N.A.	N.A.	N.A.	0.12	0.29	85.2	357.0	0.42	N.A.	0.24	N.A.	0.10	100.0
1	HZ-R_0_T_50_t_1.25_	0	50	1.25	0.12	0.33	95.1	331.8	0.36 (0.35)	0.0	0.29 (0.30)	0.0	0.10	92.8
2	HZ-R_0_T_90_t_1.25_	0	90	1.25	0.10	0.37	158.8	379.7	0.27 (0.28)	4.9	0.42 (0.41)	0.0	0.11	86.7
3	HZ-R_0_T_70_t_0.5_	0	70	0.5	0.10	0.32	114.6	322.5	0.32 (0.30)	0.2	0.36 (0.38)	0.0	0.11	95.0
4	HZ-R_0_T_70_t_2_	0	70	2	0.10	0.35	124.3	316.1	0.27 (0.28)	1.2	0.39 (0.39)	0.0	0.11	99.4
5	HZ-R_1_T_50_t_1.25_	1	50	1.25	0.12	0.33	98.2	364.8	0.37 (0.40)	0.0	0.27 (0.28)	2.3	0.10	95.9
6	HZ-R_1_T_90_t_1.25_	1	90	1.25	0.12	0.28	105.3	354.5	0.45 (0.43)	0.0	0.30 (0.31)	25.4	0.13	99.3
7	HZ-R_1_T_70_t_0.5_	1	70	0.5	0.12	0.25	105.9	342.3	0.46 (0.42)	0.0	0.31 (0.33)	50.8	0.14	94.4
8	HZ-R_1_T_70_t_2_	1	70	2	0.11	0.26	118.7	379.9	0.41 (0.40)	0.0	0.31 (0.31)	6.7	0.13	89.5
9	HZ-R_0.4_T_65_t_0.5_	0.4	65	0.5	0.11	0.37	161.1	368.9	0.30 (0.31)	11.2	0.44 (0.42)	0.0	0.13	74.5
10	HZ-R_0.5_T_50_t_0.5_	0.5	50	0.5	0.10	0.33	162.5	364.8	0.31 (0.33)	16.2	0.45 (0.44)	0.0	0.14	77.3
11	HZ-R_0.5_T_90_t_0.5_	0.5	90	0.5	0.11	0.34	145.6	329.7	0.31 (0.35)	11.0	0.44 (0.43)	0.0	0.14	81.7
12	HZ-R_0.5_T_50_t_2_	0.5	50	2	0.12	0.37	190.4	395.9	0.32 (0.32)	42.6	0.48 (0.49)	0.0	0.15	73.4
13	HZ-R_0.5_T_90_t_2_	0.5	90	2	0.10	0.32	140.8	360.1	0.32 (0.33)	1.2	0.39 (0.40)	0.0	0.12	80.8
14	HZ-R_0.5_T_70_t_1.25_	0.5	70	1.25	0.11	0.32	144.5	330.5	0.34 (0.33)	11.2	0.44 (0.45)	0.0	0.15	78.5
15	HZ-R_0.7_T_70_t_1.25_	0.7	70	1.25	0.12	0.36	150.4	379.6	0.33 (0.36)	2.0	0.40 (0.37)	0.0	0.13	85.5
16	HZ-R_0.62_T_50_t_2_	0.62	50	2	0.12	0.37	175.6	380.2	0.32 (0.32)	23.0	0.45 (0.46)	1.9	0.15	74.7
17	HZ-R_0.55_T_50_t_2_	0.55	50	2	0.12	0.31	155.9	356.5	0.38 (0.37)	5.2	0.44 (0.43)	0.4	0.17	72.3
18	HZ-R_0.1_T_75_t_1_	0.10	75	1	0.09	0.34	169.4	350.0	0.27 (0.28)	42.6	0.48 (0.46)	1.3	0.13	60.2
19	HZ-R_0.55_T_25_t_2_	0.50	25	2	0.11	0.23	103.4	332.8	0.31	N.A.	0.48	N.A.	0.15	80.0
20	0.1HZ-R_0.55_T_50_t_2_	0.55	50	2	0.12	0.25	109.5	358.9	0.48	N.A.	0.31	N.A.	0.15	91.5
21	0.3HZ-R_0.55_T_50_t_2_	0.55	50	2	0.09	0.44	162.2	327.9	0.20	N.A.	0.49	N.A.	0.10	48.7

a *R* = [TPAOH]/([NaOH] + [TPAOH]).

b *T* = desilication temperature.

c *t* = desilication time.

d *V*_micro_ = micropore volume.

e *V*_total_ = total pore volume.

f S_ext_ = external surface area.

g *S*_BET_ = surface area estimated by using the BET equation.

h HF = $\frac{V_{micro}}{V_{Total}}\times \frac{S_{ext}}{S_{\mathrm{BET}}}$ .

i χ_c_ (%) = relative crystallinity.

### 2.2 Catalyst Characterization

Porosities were estimated using N_2_ adsorption–desorption isotherms obtained from a Micromeritics ASAP 2020A analyzer. The Brunauer–Emmett–Teller (BET) method was employed to evaluate the relative mesoporous surface area (*S*_ext_/*S*_BET_) of the samples. The relative ratios of micropore-to-total pore volume (*V*_micro_/*V*_total_) were estimated using the *t*-plot method. Before the analysis, the samples were dehydrated at 90 °C for 3 h and at 300 °C for 12 h. High-resolution transmission electron microscopy (TEM, JEOL JEM-2100F CS) equipped with an Oxford X-Max 20 EDS detector was used to observe the pores created by desilication.

X-ray diffraction (XRD) patterns of the synthesized powders were obtained by using a Rigaku SmartLab diffractometer operating at 40 kV and 30 mA with a Cu Kα radiation source (λ = 1.54056 Å). Characteristic peaks of ZSM-5 ^33^ at 2θ = 7.92°, 8.80°, 14.78°, 23.18°, 23.9°, and 24.40° were used to estimate the relative crystallinity (χ_c_, eq 2), with the pristine ZSM-5 serving as the reference (χ_c_ = 100%).^34^

$$\mathrm{Relative crystallinity}\;\left(\chi_{c}\%\right)=\frac{\sum A}{\sum A_{s}}\times 100\%$$ 2

where ∑*A* is the integrated area of the diffraction peaks at 14.78°, 23.18°, 23.9°, and 24.40° and ∑*A*_s_ is the integrated area of the diffraction peaks at 14.78°, 23.18°, 23.9°, and 24.40° of parent ZSM-5 (CBV-8014).

The ^27^Al and ^29^Si MAS NMR spectra were collected on a Bruker Avance 400 at 9.4 T with spinning speeds of 10 and 8 kHz, respectively. ^27^Al spectra were acquired using a 3 μs single pulse (90°) and a 4 s delay, while ^29^Si spectra employed a 3 μs pulse and a 60 s delay. Chemical shifts were referenced to 1 M [Al­(H_2_O)_6_]^3+^ (^27^Al) and tetramethylsilane (^29^Si). All spectra were normalized to the sample weight.

Acidity was analyzed by NH_3_ temperature-programmed desorption (NH_3_-TPD, Micromeritics AutoChem II). Samples were first protonated via ion exchange with 1 M NH_4_NO_3_. A 0.20 g portion of sample was loaded into a quartz U-tube, pretreated in 2 vol % O_2_/He (50 mL min^–1^) at 500 °C for 1 h, and cooled to 150 °C under He. The sample was then exposed to 10 vol % NH_3_/He (50 mL min^–1^) for 1 h to reach adsorption equilibrium. Physisorbed NH_3_ was removed by purging with He (50 mL min^–1^) at 150 °C for 40 min. Desorption was carried out from 100 to 550 °C at 10 °C min^–1^ under He, with NH_3_ detected by a thermal conductivity detector (TCD).

### 2.3 Computational Section

Bayesian optimization (BO) was applied to iteratively refine the experimental synthesis procedure. A Gaussian process regression (GPR) surrogate with a radial basis function (RBF) kernel was implemented using Scikit-learn,^35^ and the Expected Improvement (EI)^36^ acquisition balanced exploration and exploitation. In the GPR model, three experimental parameters were used as the input features: (1) the TPAOH fraction (*R*), defined as the molar ratio of TPAOH to total hydroxide species, *R* = [TPAOH]/([NaOH] + [TPAOH]); (2) the desilication temperature (*T*, °C); and (3) the treatment time (*t*, h). These three features fully describe the experimental design space of the desilication process. The target properties (objective functions) were the micropore ratio (*V*_micro_/*V*_total_) and mesopore ratio (*S*_ext_/*S*_BET_), which were modeled as continuous responses predicted by GPR.

Because Scikit-learn minimizes by default, micro- and mesopore ratios were negated to serve as maximization objectives. Optimization was terminated when the Expected Improvement (EI) reached its minimum among the sampled conditions. The selection of the next experiment in each iteration was determined by a unified scalar acquisition function, i.e., *g*(*x*) = 0.5 EI_micro_(*x*) + 0.5 EI_meso_(*x*), to ensure that both objectives contributed equally to the BO decision while yielding a single synthesis condition per iteration. While multiobjective Bayesian optimization (MOBO) frameworks, such as Pareto-front-based methods or multiobjective expected improvement (e.g., uncertainty-aware search framework for optimizing multiple objectives),^37^ are widely used, they are suitable when each objective can be queried independently. In the present system, micropore and mesopore ratios arise from the same synthesis experiment, requiring a single unified experimental query per iteration. Therefore, a scalarized EI function was adopted instead of a full MOBO strategy, ensuring equal weighting while remaining consistent with the physical constraints of the desilication process. Thus, convergence was declared once an additional iteration failed to reduce *g*(*x*), indicating no further expected gain. Figure 1 shows the used workflow.^38^

**Figure 1:**
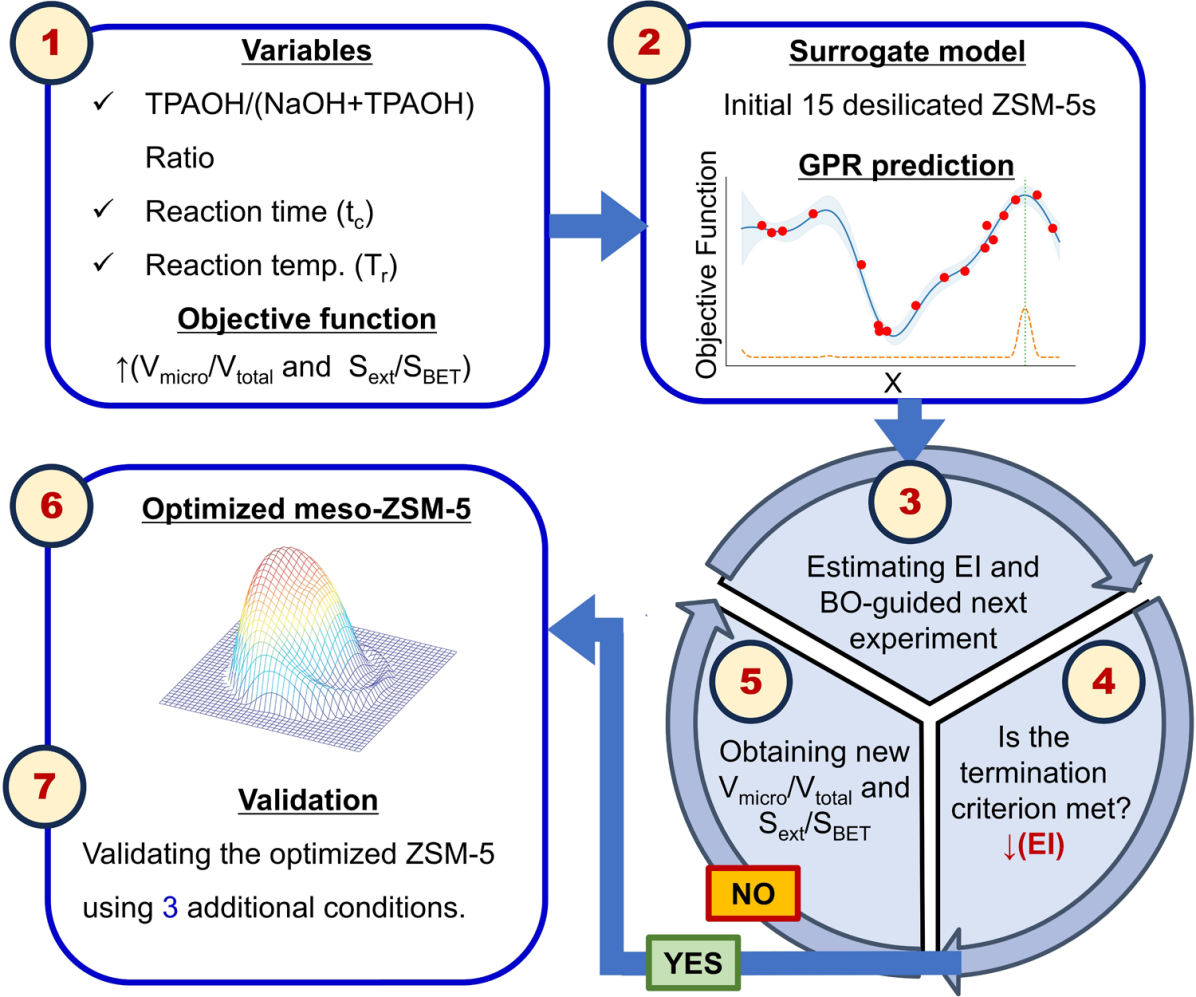
Bayesian optimization-guided workflow for catalyst design, comprising three stages: (i) experimental design (steps 1 and 2), (ii) active learning and BO cycle (steps 3–5), and (iii) validation (steps 6 and 7).

#### 2.3.1 Initial Data Set

A 15-run Box–Behnken design (BBD; Samples 1–15) provided coverage of the factor space, enabled estimation of pure error, and stabilized the surrogate model. Sample 9 (HZ-R_0.4_T_65_t_0.5_) serves as the benchmark, following the desilication procedure reported by the Pérez-Ramírez group.^22^

#### 2.3.2 Surrogate Modeling

All inputs were scaled to [−1 and 1] to avoid scale dominance and improve conditioning. GPR surrogates were trained with an RBF + WhiteKernel to capture smooth correlations and experimental noise; a Matern kernel was also tested but exhibited lower predictive accuracy. Hyperparameters were optimized by maximizing the log marginal likelihood, and predictive accuracy was evaluated by leave-one-out cross-validation (LOOCV) using *R* ^2^ and mean absolute error (MAE) as metrics.

#### 2.3.3 Optimization Loop

BO was performed with EI (via scikit-optimize). After each experiment, the data set was updated, the GPR retrained, and the next condition selected by minimizing EI. The BO iterations were terminated once the unified scalar acquisition function *g*(*x*) value reached its minimum, i.e., when the optimal condition corresponded to the lowest *g*(*x*) value among successive iterations.

#### 2.3.4 Validation

Confirmation runs were performed at a lower treatment temperature (Sample 19, HZ-R_0.55_T_25_t_2_) and using lower (0.1 M) and higher (0.3 M) OH^–^ concentrations (Samples 20 (0.1HZ-R_0.55_T_50_t_2_) and 21 (0.3HZ-R_0.55_T_50_t_2_), respectively). The consistent results obtained from this expanded search window support the optimum as being effectively global within the defined domain.

## 3 Results

The data in Figure S1 show the N_2_ isotherms, and Table 1 displays the porosities of tested ZSM-5s. The pristine ZSM-5 (HZ-C) displayed type I isotherms with an H3 type hysteresis loop. The mesopore surface area (*S*_meso_ = 85.2 m^2^ g^–1^), total surface area (*S*_total_ = 357.0 m^2^ g^–1^), micropore volume (*V*_micro_ = 0.12 m^3^ g^–1^), and total pore volume (*V*_total_ = 0.29 m^3^ g^–1^) were close to the reported data.^39^ The estimated HF value was 0.10.

NaOH-treated (Samples 1–4) and TPAOH-treated (Samples 5–8) ZSM-5s displayed type I-like isotherms similar to HZ-C, with slightly higher adsorption at *P*/*P*_0_ > 0.8, reflecting increased mesopore contributions. H3 hysteresis loops, typical of slit-like pores between agglomerated particles, were observed. These results suggest that either NaOH or TPAOH alone produces only limited intracrystalline mesoporosity.

Closer inspection reveals distinct effects of the desilication agents. NaOH led to lower micropore ratios (0.27–0.36), indicating significant framework damage, whereas TPAOH preserved microporosity (0.40–0.46), close to HZ-C (0.42), while inducing new micropores. Among Samples 1–8, the highest HF (0.14) was achieved for Sample 7; however, this value correlated weakly with mesoporosity, reflecting its low mesopore ratio (0.31) but high micropore ratio (0.46).

ZSM-5 samples treated with (NaOH + TPAOH) exhibited type IV isotherms with H4 hysteresis loops, confirming enhanced mesoporosity. Sample 9, prepared under the same desilication condition as the benchmark,^22^ showed *S*_meso_ = 161.1 m^2^ g^–1^, *S*_total_ = 368.9 m^2^ g^–1^, *V*_micro_ = 0.11 m^3^ g^–1^, and *V*_total_ = 0.37 m^3^ g^–1^, giving an HF value of 0.13 with micropore and mesopore ratios of 0.30 and 0.44, respectively. Notably, nearly all (NaOH + TPAOH)-treated ZSM-5s (Samples 9–15) achieved higher HF values (0.12–0.17), characterized by increased mesopore ratios (0.39–0.49) and slightly reduced micropore ratios (0.31–0.35), compared with those treated solely with NaOH or TPAOH.

The pore-size distribution, derived from the desorption branch using the Barrett–Joyner–Halenda (BJH) method, is shown in Figure S2. For ZSM-5 treated with either NaOH (Samples 1 and 4) or TPAOH alone (Samples 5 and 8), mesopore formation was negligible within 2–30 nm. In contrast, mixed NaOH/TPAOH treatments (Samples 9 and 15) generated a broad mesopore distribution spanning ∼2–30 nm.

The data in Figure S3 show the XRD patterns of desilicated ZSM-5s, and Table 1 lists their χ_c_ values. For NaOH- and TPAOH-treated samples, most χ_c_ values exceeded 90%, except Sample 2 (86.7%) at the highest desilication temperature (90 °C) and Sample 8 (89.5%) at the longest treatment time (2 h). In contrast, (NaOH + TPAOH)-treated samples exhibited lower crystallinities (73.4–85.5%), indicating stronger desilication by the mixed solution. For example, at 70 °C, Samples 14 and 15 showed lower χ_c_ values (78.5% and 85.5%) than Samples 4 (99.4%) and 8 (94.4%), despite shorter treatment times. These results confirm that the mixed solution is more effective than either NaOH or TPAOH alone as a desilication agent.

The data in Figure 2 (a and b) show the leave-one-out cross-validation (LOOCV) results for the initial data set (Samples 1–15). LOOCV, a special case of cross-validation in which each fold is trained on *n* – 1 samples and tested on the held-out sample,^40,41^ was used to assess model generalizability. The close agreement between measured and GPR-predicted values for both micropore and mesopore ratios confirms that the 15 samples provide a sufficient basis for constructing a reliable surrogate model.

**Figure 2:**
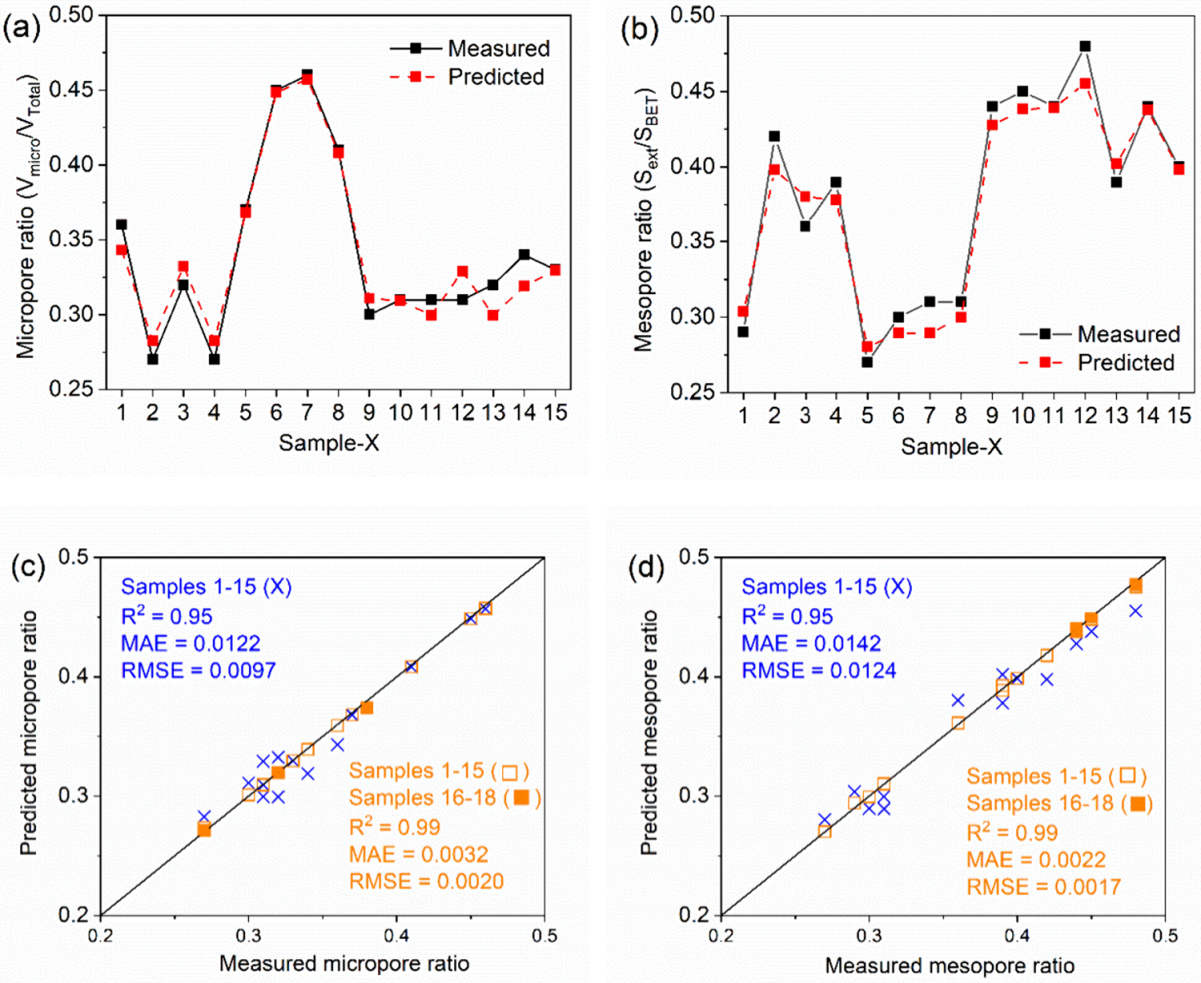
LOOCV results showing actual vs predicted values of Samples 1 to 15 for (a) micropore ratio (*V*_micro_/*V*_total_) and (b) mesopore ratio (*S*_meso_/*S*_BET_). Performance of the summation model using 15 (Samples 1–15) and 18 data points (Samples 1–18) for (c) micropore ratio (*V*_micro_/*V*_total_) and (d) mesopore ratio (*S*_meso_/*S*_BET_).

Parity plots of actual versus GPR-predicted micropore and mesopore ratios for Samples 1–15 are given in Figure 2 (c and d). Before optimization, the parity plots for the 15 samples achieved high accuracy, with *R* ^2^ values of 0.95 and similar MAE (0.0122, 0.0142) and RMSE (0.0097, 0.0124) for micropore and mesopore ratios, respectively. These results confirm that the surrogate reliably captures the dependence of the objective functions on the input parameters.

The LOOCV and parity plot results of the initial 15 samples confirm the reliability of the GPR surrogate model for subsequent active learning. As shown in Table S1, the evolution of the EI values (high → low → rebound), together with the ranking of the top five candidate points generated for Samples 16–18, clearly indicates that the Bayesian optimization locally converged within 3 iterations. These top-5 EI candidates illustrate how the model progressively reduced uncertainty. Such efficiency has also been reported by Ohyama et al.^42^ and Tian et al.,^43^ who demonstrated that only 2 and 3 BO iterations, respectively, were sufficient to reach their optimal conditions. After three iterations (Samples 16–18), Sample 17 was identified as the optimum, exhibiting the lowest EI values for both micro- (5.2) and mesopore (0.4) ratios (Table 1). Its micropore ratio (0.38) and mesopore ratio (0.44) yielded the highest HF value (0.17) among desilicated ZSM-5s, with χ_c_ = 72.3%. The corresponding N_2_ isotherms (Figure S4), BJH pore distributions (Figure S5), and XRD patterns (Figures S6) of Samples 16–18 were shown in the Supporting Information.

The efficacy of BO-guided active learning was validated by parity plots of actual versus GPR-predicted micropore and mesopore ratios, incorporating three additional samples from the BO iterations (Samples 1–18, Figure 2 (c and d)). The *R* ^2^ values improved (0.99 and 0.99), while MAE (0.0032, 0.0022) and RMSE (0.0020, 0.0017) decreased. Calibration curves (Figure S7) further showed that miscalibration areas declined from 0.0307 to 0.0294 (micropore) and 0.0055 to 0.0034 (mesopore), with calibration errors reduced from 0.1164 to 0.1050 and 0.0898 to 0.0722, respectively. As shown in Table S2, these accuracy improvements collectively demonstrate that the BO–GPR framework progressively refined the surrogate model and reduced both prediction error and uncertainty throughout the optimization. These results demonstrate that the BO–GPR framework effectively lowers prediction error and uncertainty.^44^

Samples 19 (HZ-R_0.5_T_25_t_2_), 20 (0.1HZ-R_0.55_T_50_t_2_), and 21 (0.3HZ-R_0.55_T_50_t_2_) were synthesized outside the surrogate range to validate the BO-optimized condition. Sample 19 was treated at room temperature, while Samples 20 and 21 varied OH^–^ concentration (0.1 and 0.3 M vs 0.2 M for Samples 1–18). Sample 20 exhibited a higher micropore ratio (0.48) and lower mesopore ratio (0.31) than Sample 17 (0.38, 0.44), whereas Samples 19 and 21 showed the opposite trend. All three yielded lower HF values (0.10–0.15) than the optimal Sample 17 (0.17). High χ_c_ values of Samples 19 (80.0%) and 20 (91.5%) reflected limited desilication under milder conditions, while the low χ_c_ of Sample 21 (48.7%) indicated severe framework degradation at a higher OH^–^ concentration. The optimum (HZ-R_0.55_T_50_t_2_, Sample 17) was comparatively analyzed with the pristine (HZ-C) and benchmark (HZ-R_0.4_T_65_t_0.5_, Sample 9) to reveal their differences in physicochemical properties.

Transmission electron microscopy images of HZ-C, the benchmark sample, and the optimized Sample 17 are shown in Figure 3. The variation in image contrast thus reflects differences in local electron density, where darker regions denote intact zeolitic frameworks and brighter regions indicate silica removal during controlled desilication. The pristine HZ-C exhibited compact, sharp-edged crystals with uniformly dark contrast, indicating dense microporous domains and the absence of internal voids. In contrast, both desilicated samples displayed regions of lighter contrast corresponding to intracrystalline mesopores of approximately 5–10 nm, confirming successful mesopore formation.

**Figure 3:**
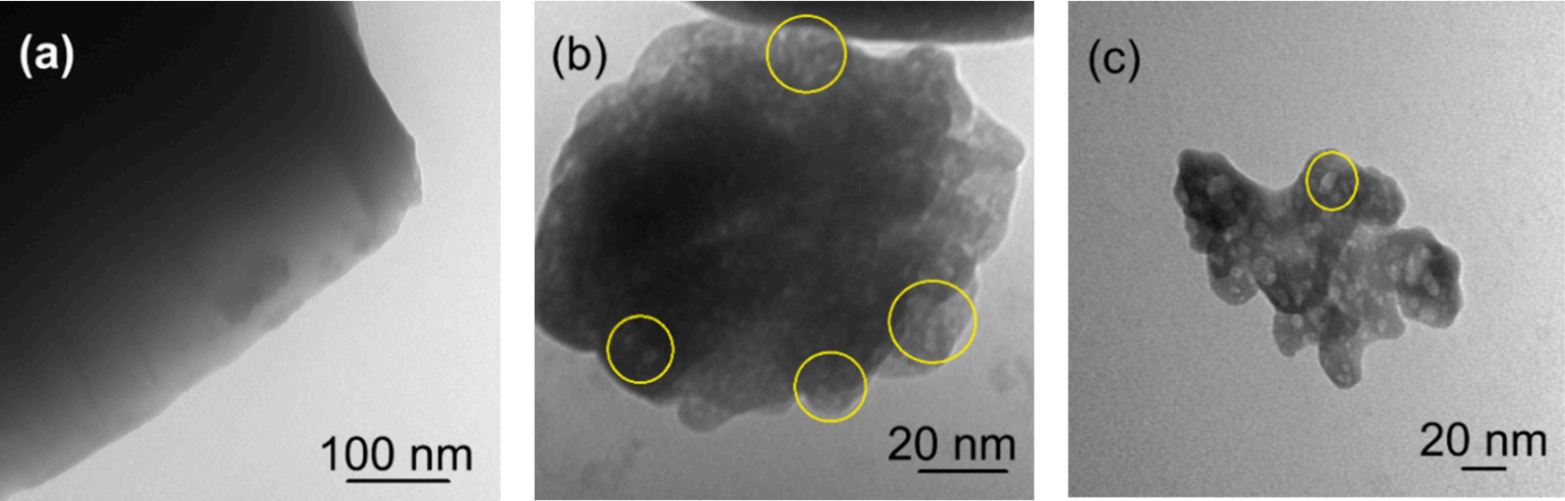
TEM images of (a) HZ-C, (b) Sample 9 (benchmark), and (c) Sample 17 (optimum). Desilication-created mesopores are highlighted in yellow circles.

^27^Al and ^29^Si MAR NMR spectra of HZ-C, Sample 9 (HZ-R_0.4_T_65_t_0.5_), and Sample 17 (HZ-R_0.55_T_50_t_2_) are given in Figure 4. In the ^27^Al spectra, a sharp resonance at ∼54 ppm (framework Al­(IV)) and a weak signal at ∼0 ppm (amorphous Al­(VI)) appeared.^45^ Samples 9 and 17 exhibited stronger Al­(IV) and no detectable Al­(VI), suggesting removal of amorphous Al­(VI) and possible realumination of framework Al during desilication.^22^

**Figure 4:**
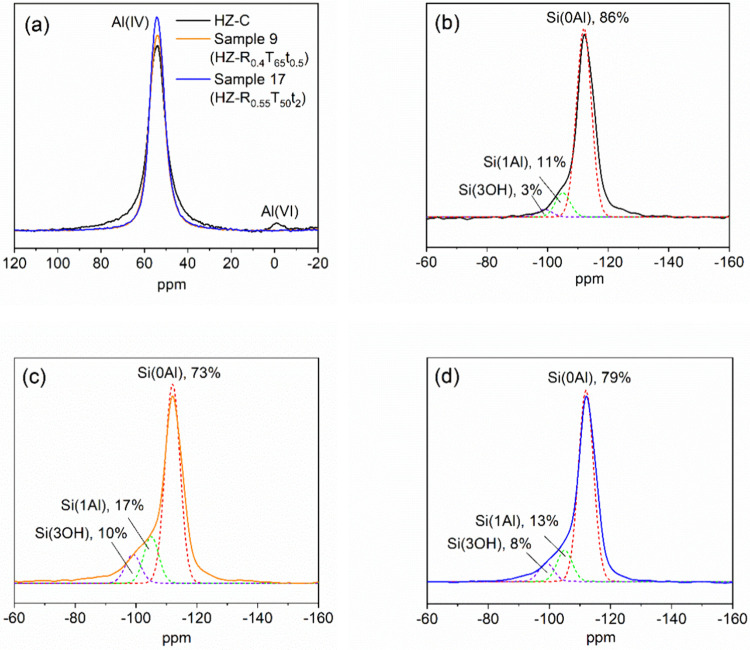
(a) ^27^Al MAS NMR spectra of HZ-C, Sample 9 (benchmark), and Sample 17 (optimum). ^29^Si MAR NMR spectra of (b) HZ-C, (c) Sample 9 (benchmark), and (d) Sample 17 (optimum).

The ^29^Si spectra were deconvoluted into Si­(0Al) (−112 ppm), Si­(1Al) (−105 ppm), and Si­(3OH) (−99 ppm).^26^ The Si­(0Al) fraction decreased from 86% (HZ-C) to 79% (Sample 17) and 73% (Sample 9), with Si­(1Al) and Si­(3OH) increasing correspondingly; Si­(3OH) rose from 3% (HZ-C) to 8% (Sample 17) and 10% (Sample 9). The framework Si/Al ratio^46^ declined from 17.4 (HZ-C) to 13.0 (Sample 17) and 9.2 (Sample 9), indicating reduced Si–O–Si coordination, increased Si–O–Al bonding, and increased silanol concentration after desilication. The harsher desilication conditions of the benchmark material lowered the framework Si/Al ratio compared to the optimum.

The NH_3_-TPD profiles of HZ-C, the benchmark sample, and the optimal Sample 17 are shown in Figure 5. HZ-C exhibits two desorption peaks at ∼180 and 370 °C with a total NH_3_ uptake of 0.52 mmol g^–1^. Sample 9 showed merged peaks with reduced uptake (0.26 mmol g^–1^), while Sample 17 retained a similar profile to HZ-C but with a much lower intensity (0.07 mmol g^–1^). While NH_3_-TPD provides only qualitative insight into acidity and is often considered unreliable for precise acid quantification,^47^ the observed trend indicates that desilication markedly modifies the acid distribution.

**Figure 5:**
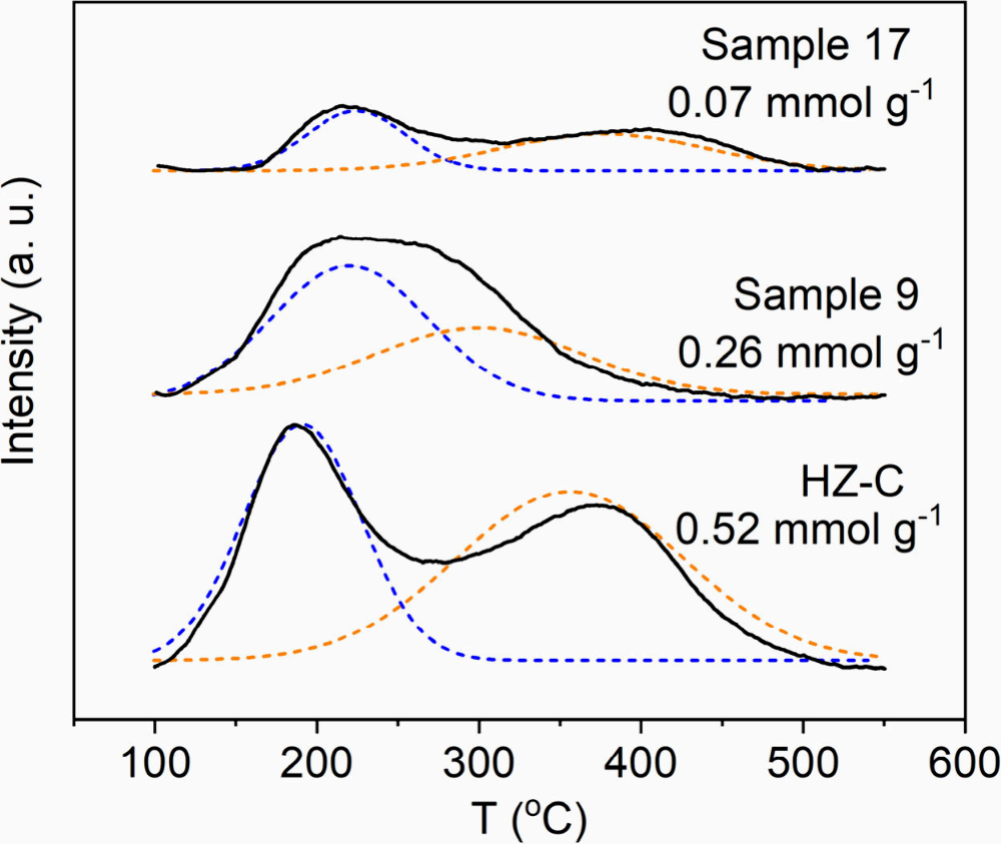
NH_3_-TPD profiles of HZ-C, Sample 9 (benchmark), and Sample 17 (optimum).

Sensitivity analysis (Figure S8) ranked desilication variables as *R* (0.15) > *T* (0.13) > *t* (0.07) for micropores and *R* (0.13) > *T* (0.10) > *t* (0.06) for mesopores, highlighting the dominant role of *R* and the minor effect of *t* in pore development of desilicated ZSM-5.

## 4 Discussion

Differences in porosity and crystallinity revealed distinct desilication behaviors. NaOH induced aggressive desilication, as OH^–^ promoted silicon hydrolysis and framework dissolution, leading to micropore collapse and irregular mesopores.^48,49^ In contrast, TPAOH moderated desilication: bulky TPA^+^ cations blocked OH^–^ access to micropores, yielding controlled Si extraction, and preserved microporosity.^22,49,50^ However, TPAOH alone provided limited mesoporosity improvement despite maintaining micropores.

The NaOH–TPAOH mixture enhanced mesoporosity while sacrificing some microporosity. TPA^+^ shielded channels from OH^–^ erosion, whereas NaOH drove strong mineralization and over-desilication.^51,52^ The optimized condition (*R* = 0.55, *T* = 50 °C, *t* = 2 h) closely matched the benchmark (*R* = 0.40, *T* = 65 °C, *t* = 0.5 h), yet yielded higher microporosity (0.38 vs 0.30) at the same mesoporosity (0.44).

Desilication conditions affect not only porosity but also acidity of hierachical ZSM-5s. The comparative analysis of pristine, the benchmark, and the optimum samples showed that realumination and silanol generation occurred during desilication, resulting in varying acidities.

The characterization results show that minor parameter shifts can strongly affect the hierarchy and acidity. Sensitivity analysis further identified *R* and *T* as dominant factors, underscoring that one-variable-at-a-time approaches cannot capture such nuances and highlighting the necessity of active-learning-guided optimization.

Figure 6 shows the HF contour plot as a function of micropore and mesopore ratios, comparing CBV-8014, Sample 9, Sample 17 (optimum), and selected catalysts of recent studies.^53–56^ Both Liao et al.^53^ and this work achieved the highest HF (∼0.17), but with distinct pore distributions: Liao and co-workers’ sample had a higher mesopore ratio (0.50) but lower micropore ratio (0.34), while this work yielded 0.38 and 0.44, respectively. The difference arises from pure 0.1 M NaOH treatment in the former, which enhanced the mesoporosity at the cost of micropores.

**Figure 6:**
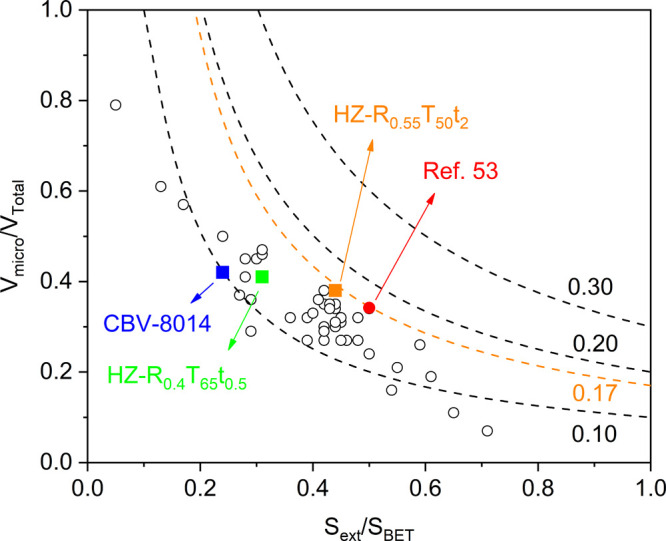
HF contour plot as a function of micropore and mesopore ratios by using CBV-8014, the benchmark, the optimum (HZ-R_0.55_T_50_t_2_), and selected studies of recent works.

Notably, Liao et al.’s^53^ best ZSM-5 for 4-*n*-propylphenol dealkylation, treated with 0.2 M NaOH, showed a lower HF (∼0.14) but higher activity, attributed to stronger desilication-induced LAS (relocated Al^3+^) than the original BAS. The distribution and relocation of BAS—within the same 10-ring or across neighboring rings—also influence coke precursor formation and catalyst lifetime.^57^ Hence, a higher HF does not necessarily yield better activity. Future work should combine BO-guided optimization with a detailed physicochemical analysis to tailor desilicated ZSM-5 for specific reactions.

## 5 Conclusions

A Bayesian optimization-guided framework was used to direct the desilication of ZSM-5, achieving hierarchical structures with favored micro–mesopore balance. The optimal conditions (*R* = 0.55, *T* = 50 °C, *t* = 2 h) produced the highest HF (0.17) among all samples, combining enhanced mesoporosity with retained microporosity. Characterization revealed that NaOH and TPAOH play complementary roles—NaOH drives Si extraction, while TPA^+^ moderates framework dissolution—yielding controlled mesopore formation and maintained crystallinity. NMR and acidity analyses showed realumination and silanol generation, reflecting a modified acid site distribution after desilication. Sensitivity analysis identified the TPAOH fraction and treatment temperature as key variables influencing the hierarchical evolution. The BO–GPR strategy effectively reduced experimental demand and prediction uncertainty, offering a generalizable, data-driven approach for accelerating catalyst design and optimizing complex synthesis spaces in zeolite engineering.

## Supplementary Material



## Data Availability

Data will be made available on request. Python code of this study can be downloaded through https://github.com/jimmy910603/BO-with-hierachy-factor.

## Notes

N_2_ isotherms and porosities; XRD patterns; calibration curves; sensitivity analysis results (PDF)

## Abbreviations

BO: Bayesian optimization
HF: Hierarchical factor
RSM: Response surface methodology
*R*: The ratio of tetraethylammonium hydroxide (TPAOH) to the mixed sodium hydroxide (NaOH) and TPAOH solution [TPAOH]/([NaOH] + [TPAOH])
*T*: Desilication temperature (°C)
*t*: Desilication time (h)
EI: Expected improvement
GPR: Guassian process regression
LOOCV: Leave-one-out cross-validation
MAE: Mean absolute error
MOBO: Multiobjective Bayesian optimization
RBF: Radial basis function
RMSE: Root mean squared error
