# Supplementary material for: Accelerating Hierarchical ZSM‑5 Engineering via Bayesian Optimization-Guided Discovery
Source: ACS Mater Au. 2025 Dec 22;6(2):415–24. doi: 10.1021/acsmaterialsau.5c00196 (PMC12983102; doi:10.1021/acsmaterialsau.5c00196)
Supplement: Supplementary file 1 [file mg5c00196_si_001.pdf]

Supporting Information

Accelerating Hierarchical ZSM-5 Engineering via Bayesian Optimization-Guided Discovery

Tzu-Hung Wen,[†] Cheng-Yi You,[†] Ting-Hao Liu,[†] Bryan R. Goldsmith,[‡] and Yu-Chuan Lin[†,§,*]

[†] Department of Chemical Engineering, National Cheng Kung University, Tainan 70101, Taiwan

[‡] Department of Chemical Engineering, University of Michigan, Ann Arbor, Michigan 48109-2136, United States of America

[§] Center for Resilience and Intelligence on Sustainable Energy Research (RiSER), National Cheng Kung University, Tainan 70101, Taiwan

*Corresponding author's Email: yclin768@mail.ncku.edu.tw (Y.-C. Lin)

Number of pages: 11

Number of figures: 8

Number of tables: 2

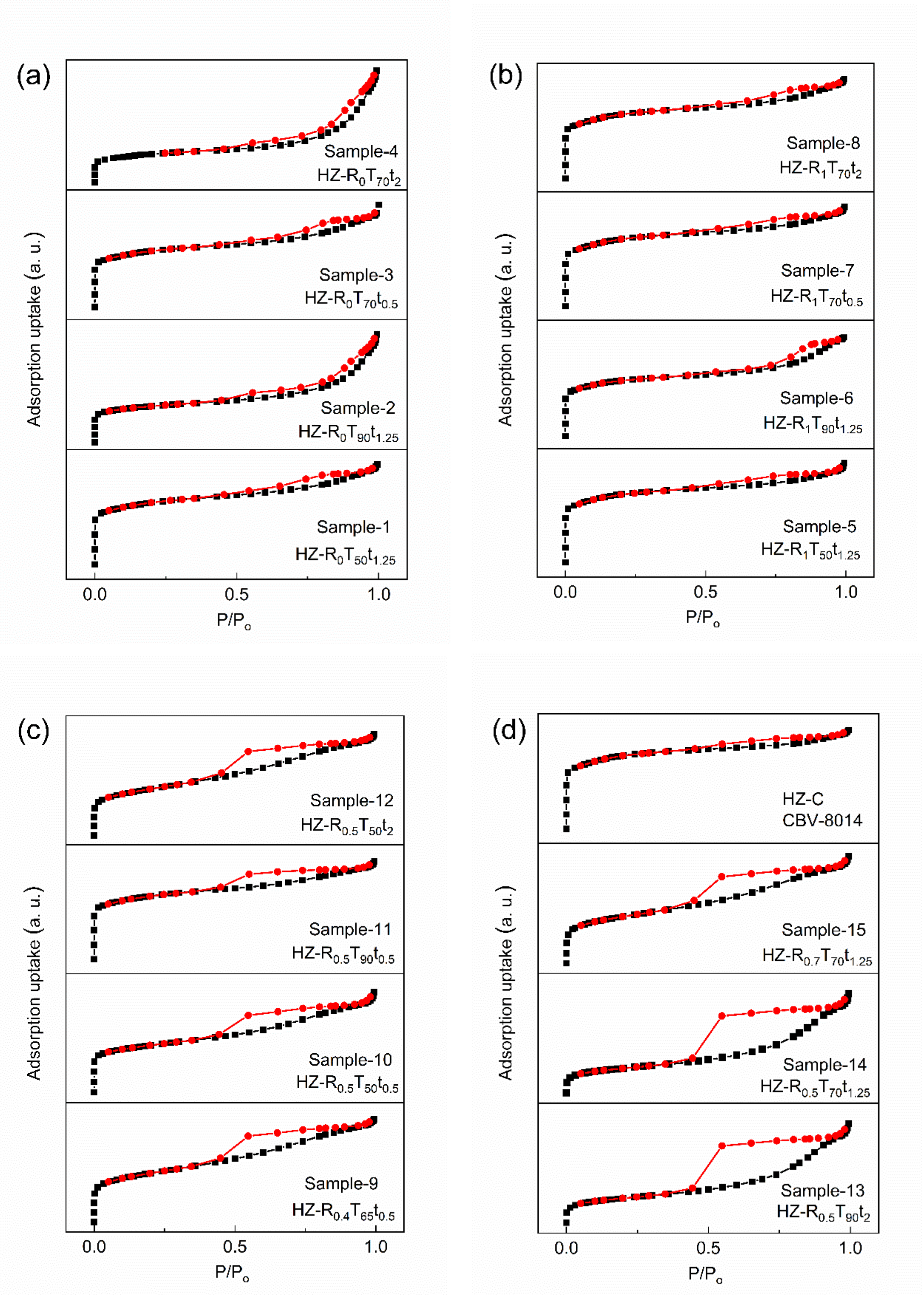

Figure S1. N_2 adsorption (black) and desorption (red) isotherms of (a) NaOH-treated, (b) TPAOH-treated, and (c and d) (NaOH + TPAOH) treated ZSM-5s and CBV-8014 (HZ-C). P_0 indicates the saturation vapor pressure of nitrogen at 77 K.

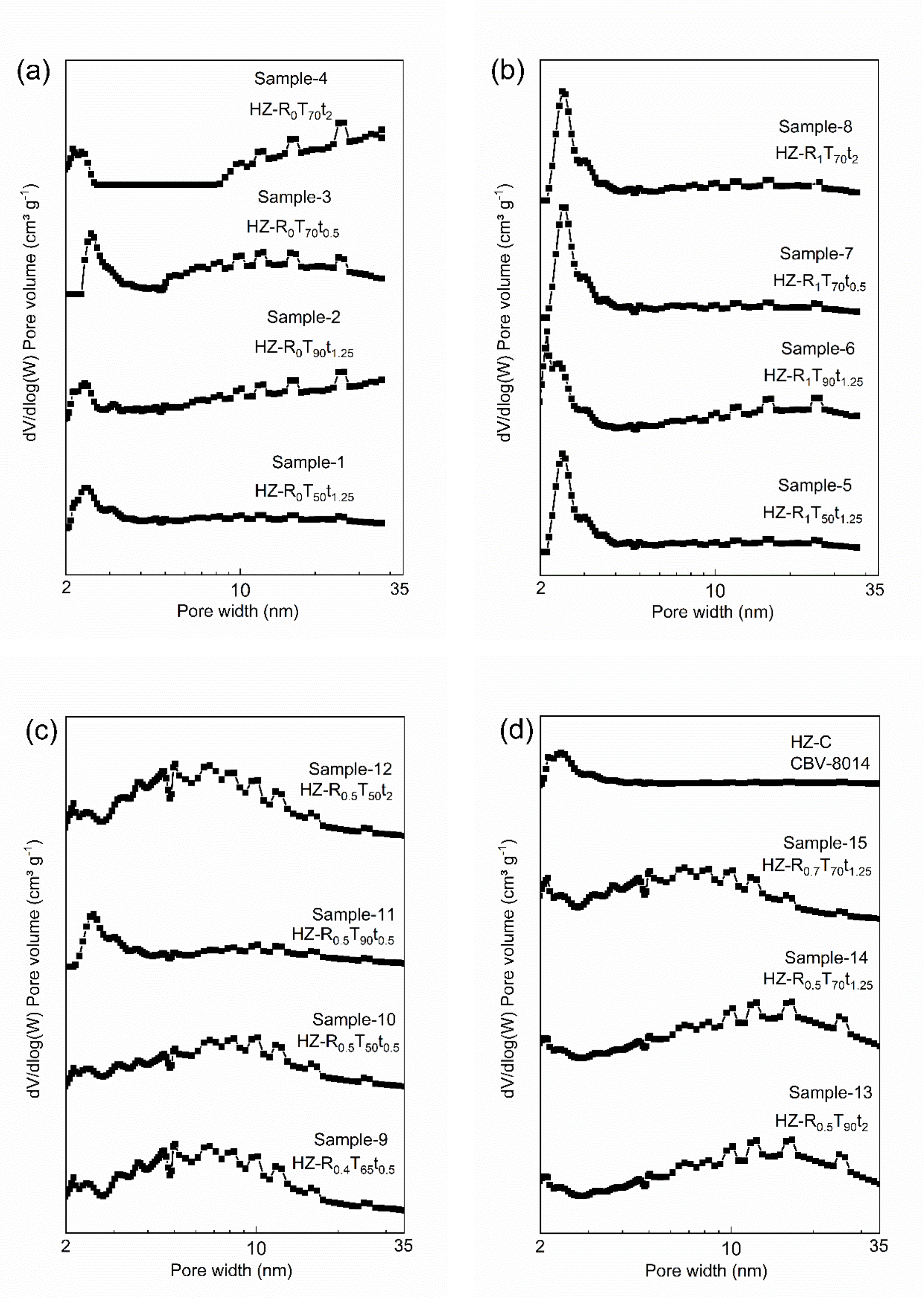

Figure S2. BJH pore size distributions of (a) NaOH-treated, (b) TPAOH-treated, and (c and d) (NaOH + TPAOH) treated ZSM-5s and CBV-8014 (HZ-C).

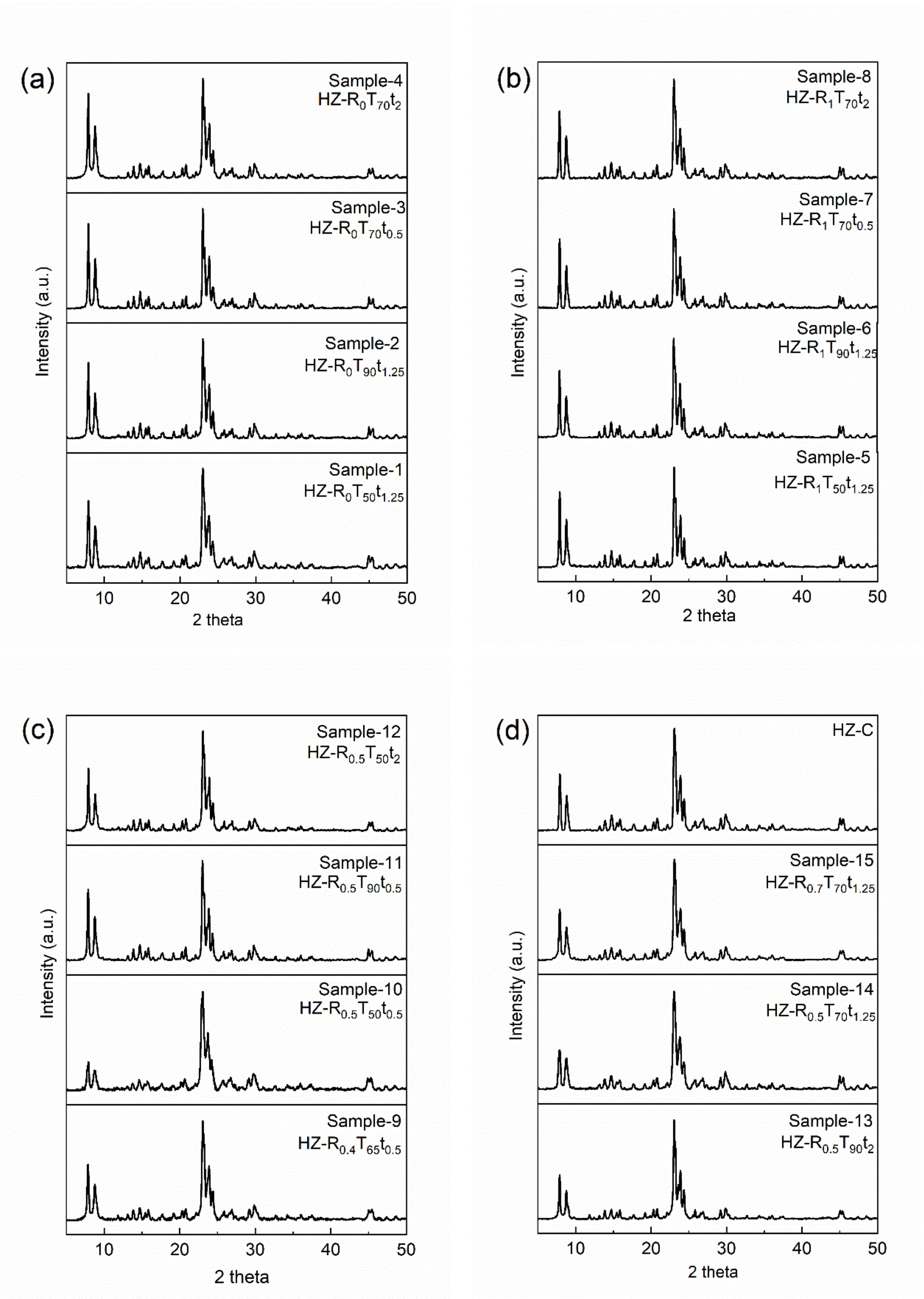

Figure S3. XRD patterns of (a) NaOH-treated, (b) TPAOH-treated, and (c and d) (NaOH + TPAOH) treated ZSM-5s and CBV-8014 (HZ-C).

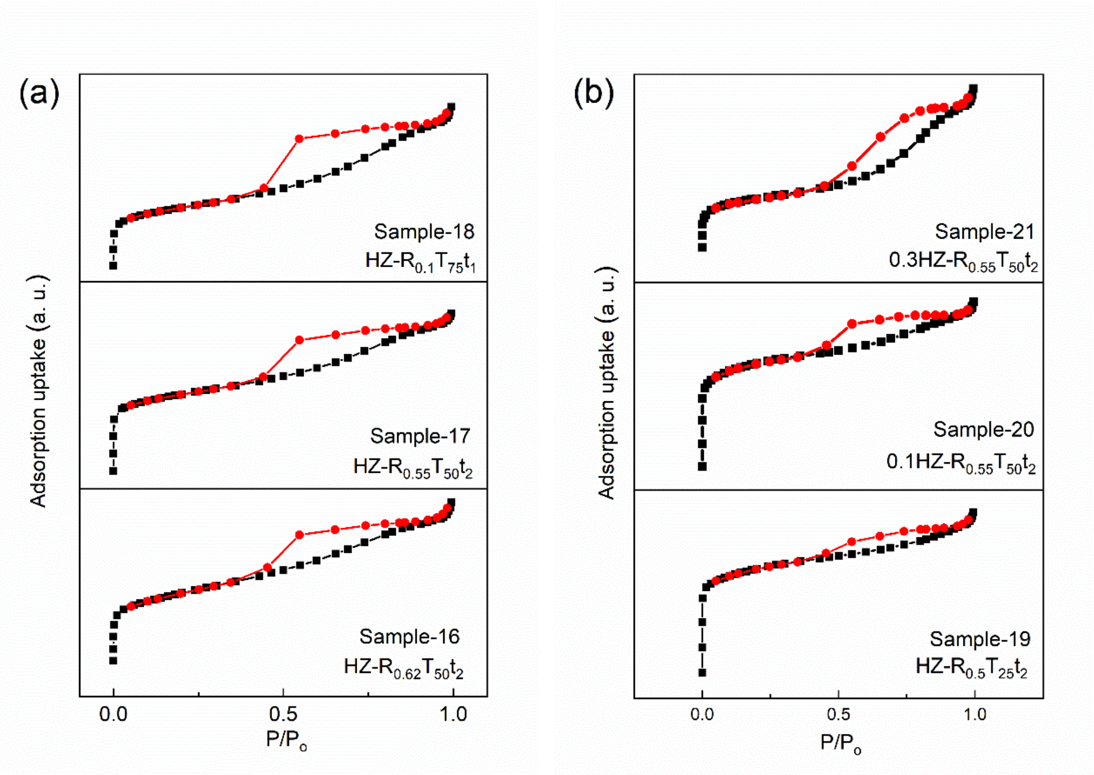

Figure S4. N_2 adsorption (black) and desorption (red) isotherms of (a) BO-iterated samples (Samples 16–18) and (b) validation samples (Samples 19–21). P_0 indicates the saturation vapor pressure of nitrogen at 77 K.

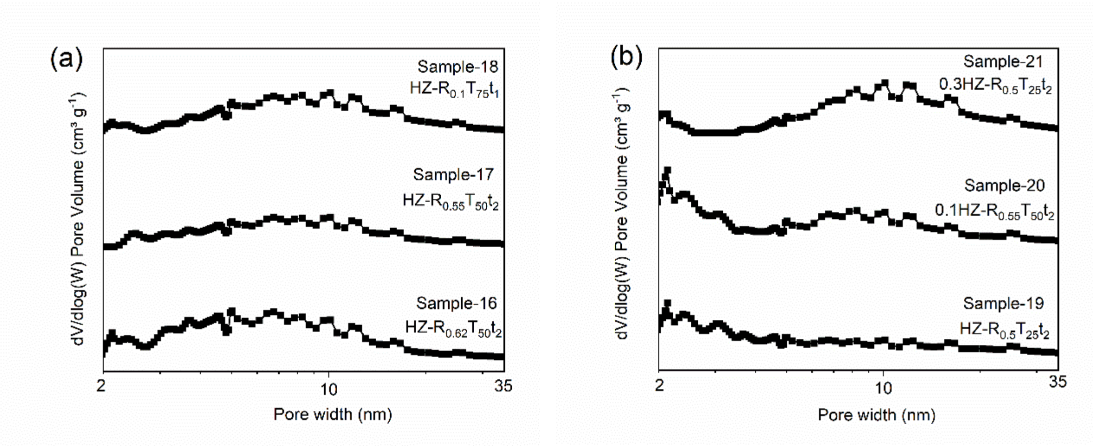

Figure S5. BJH pore size distributions of (a) BO-iterated samples (Samples 16–18) and (b) validation samples (Samples 19–21).

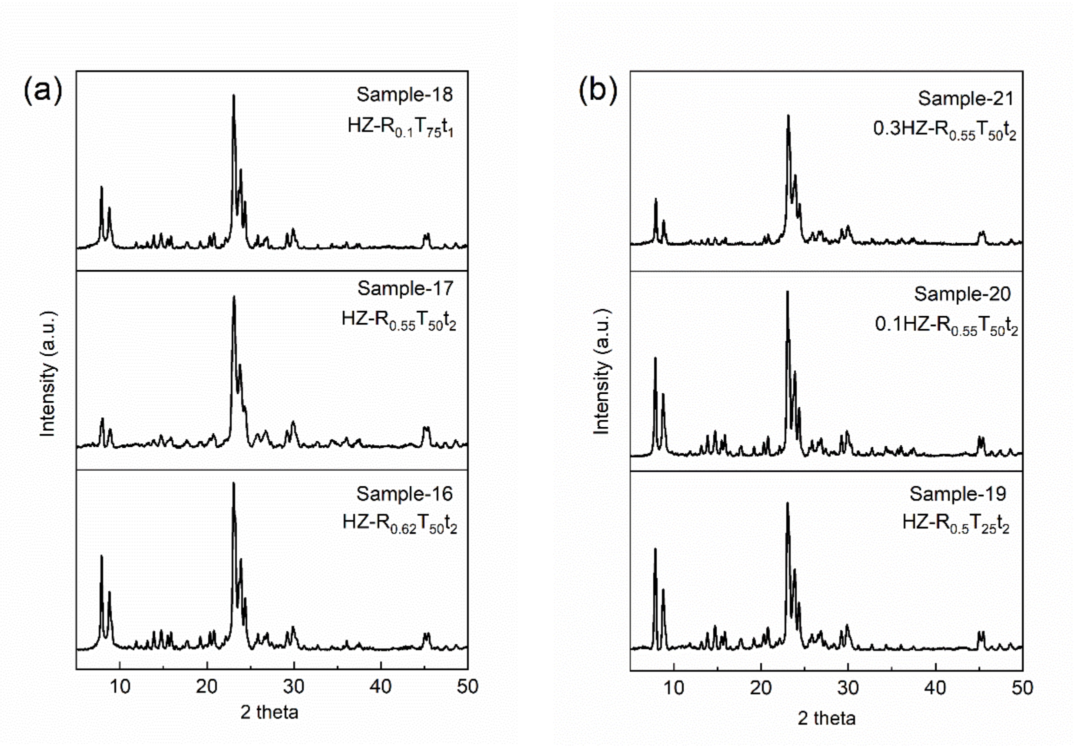

Figure S6. XRD patterns of (a) BO-iterated samples (Samples 16–18) and (b) validation samples (Samples 19–21).

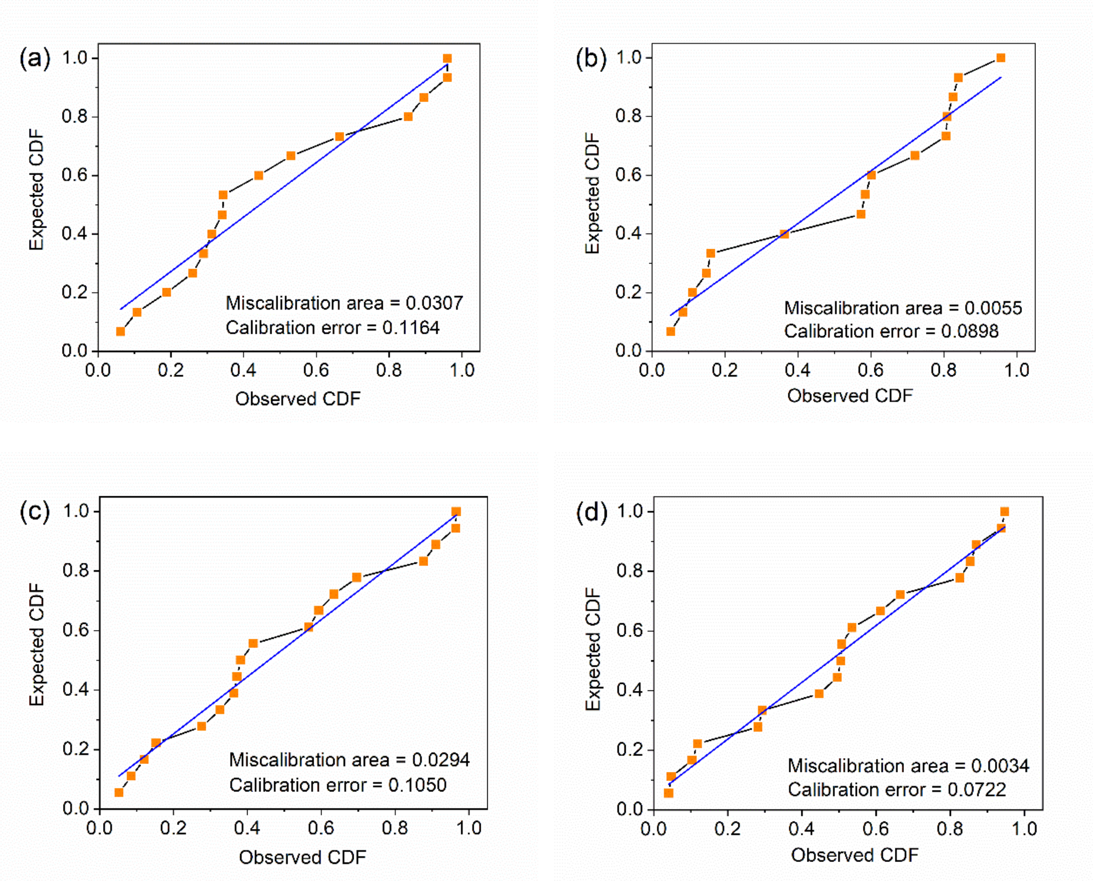

Figure S7. Calibration curves derived from the (a) micropore and (b) mesopore ratios of the initial dataset (Samples 1–15), and from the (c) micropore and (d) mesopore ratios of the total dataset (Samples 1–18). CDF = cumulative distribution function. Diagonal blue line denotes parity between the observed and expected CDFs.

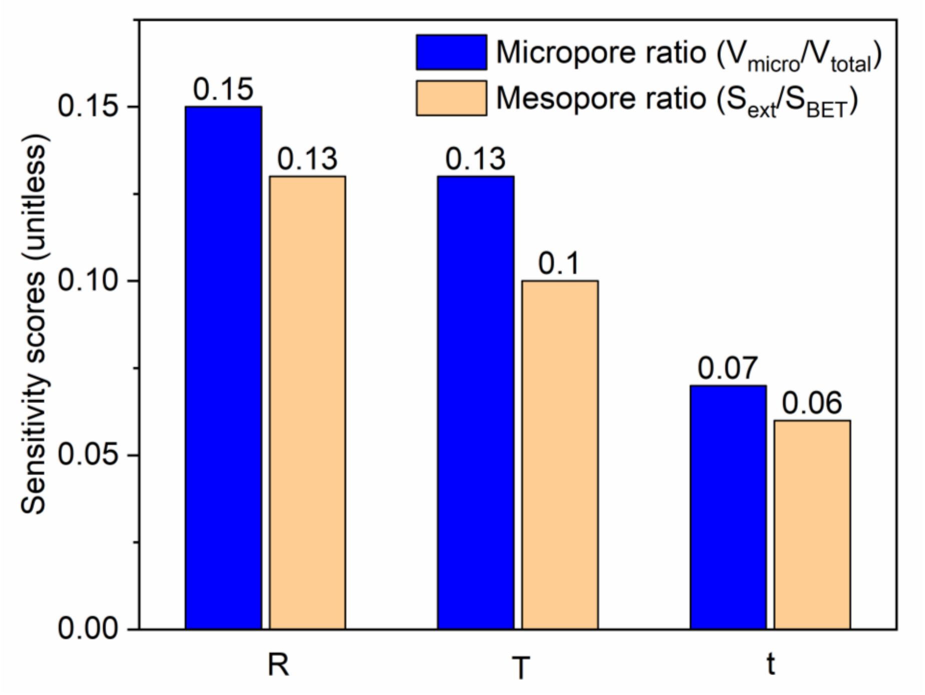

Figure S8. Scores representing normalized changes in the objective functions, that is, micropore (V_{micro}/V_{total}) and mesopore (S_{ext}/S_{BET}) ratios, in response to the variables (R, T, and t).

Identification of Bayesian Optimization Convergence Through EI Evolution

As summarized in **Table S1**, the EI evolution indicates that Bayesian optimization converged within iterations 16–18. Sample 16 exhibited elevated EI values (EI_{micro} = 23.0–35.2; EI_{meso} = 1.8–8.9) owing to the uncertainty associated with the initial dataset, prompting BO to prioritize exploration. After incorporating Sample 17, EI values decreased markedly (EI_{micro} = 5.2–7.8; EI_{meso} = 0.4–3.4), reflecting reduced model uncertainty and improved surrogate accuracy. In contrast, Sample 18 showed a rebound in EI (EI_{micro} = 42.6–52.2; EI_{meso} = 1.3–3.2) as the model probed sparsely sampled regions. This characteristic "high → low → rebound" profile signals local convergence. Accordingly, the BO-guided synthesis condition of Sample 17 is identified as a local optimum point.

Table S1. The top 5 EI values of Samples 16, 17, and 18 identified through BO.

Sample	R	T	t	EI_{micro}	EI_{meso}
	0.55	60	2	35.2	1.8
	**0.62**	**50**	**2**	**23.0**	**1.9**
16	0.50	60	2	34.0	8.9
	0.60	60	2	30.9	4.2
	0.60	55	2	29.8	3.9
	0.40	85	2	7.4	1.2
	0.50	70	2	7.8	3.4
17	0.50	80	2	6.4	2.1
	**0.55**	**50**	**2**	**5.2**	**0.4**
	0.60	70	2	5.8	1.3
	0.3	50	2	47.2	1.4
	0.3	80	2	50.8	3.0
18	0.7	75	1	52.2	3.2
	0.8	80	2	45.5	2.1
	**0.1**	**75**	**1**	**42.6**	**1.3**

Progressive Improvement and Convergence of BO-GPR Predictive Accuracy

As shown in **Table S2**, both the micropore and mesopore GPR models show clear improvements in predictive accuracy from Samples 16 to 18. R^2 increases from 0.96 to 0.99, while MAE and RMSE decrease by more than 60–70%, indicating that each BO-selected experiment substantially reduced model uncertainty. By Sample 18, the prediction errors approached the noise level of the physisorption measurements, demonstrating that the surrogate models had effectively converged. This accuracy evolution supports the conclusion that the BO process reached its optimal region at Sample 17, with 3 iterations representing boundary exploration rather than further improvement.

Table S2. BO-GPR Predictive Accuracy of Micropore and Mesopore Ratios

Sample	R^2		MAE		RMSE	
	micro	meso	micro	meso	micro	meso
16	0.96	0.96	0.0116	0.0132	0.0084	0.0069
17	0.97	0.97	0.0082	0.0073	0.0043	0.0035
18	0.99	0.99	0.0032	0.0022	0.0020	0.0017
